# Supplementary material for: Novel Method for Electroless Etching of 6H–SiC
Source: Nanomaterials (Basel). 2020 Mar 17;10(3):538. doi: 10.3390/nano10030538 (PMC7153389; doi:10.3390/nano10030538)
Supplement: Supplementary file 1 [file nanomaterials-10-00538-s001.pdf]

SUPPLEMENTARY MATERIALS

Novel Method for Electroless Etching of 6H–SiC

Gyula Károlyházy [1,*], Dávid Beke [1], Dóra Zalka [1], Sándor Lenk [2], Olga Krafcsik [2], Katalin Kamarás [1] and Ádám Gali [1,2,*]

[1] Institute for Solid State Physics and Optics, Wigner Research Centre for Physics, Konkoly-Thege Miklós út 29-33, H-1121 Budapest, Hungary; beke.david@wigner.hu (D.B.), zalka.dora@wigner.hu (D.Z.), kamaras.katalin@wigner.hu (K.K.), gali.adam@wigner.hu (A.G.)

[2] Department of Atomic Physics, Budapest University of Technology and Economics, Budafoki út 8, H-1111 Budapest, Hungary; lenk@eik.bme.hu (S.L.), krafcsik@eik.bme.hu (O.K.)

* Correspondence: karolyhazy.gyula@wigner.hu, gali.adam@wigner.hu

Characterizing the initial 6H-SiC sample

For characterizing the as-purchased initial SiC material we used Raman spectroscopy (**Figure S1**), XRD (not shown), and SEM-EDX (**Figure S2**). Raman and XRD confirmed the hexagonal (6H) polytype of SiC and as for dopants, SEM-EDX detected stochiometric presence of carbon and silicon accompanied by no additional elements, so the doping levels must be below the detection level of SEM-EDX.

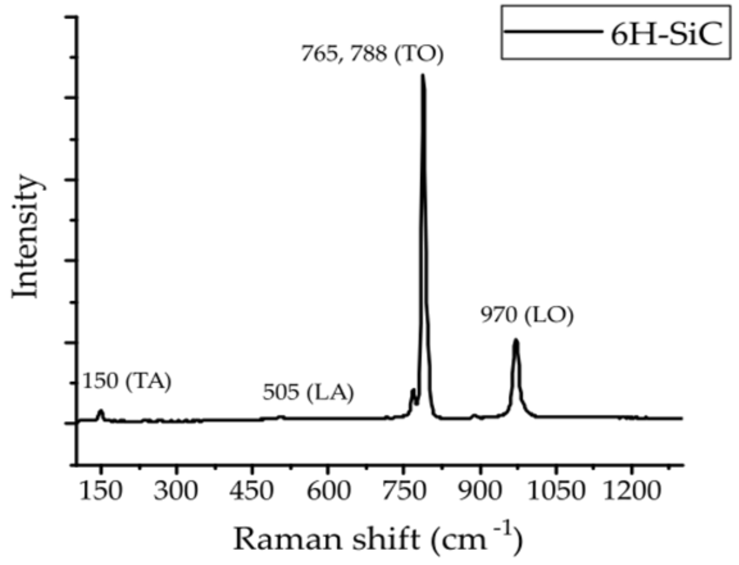

Figure S1. Raman spectrum of the initial bulk material

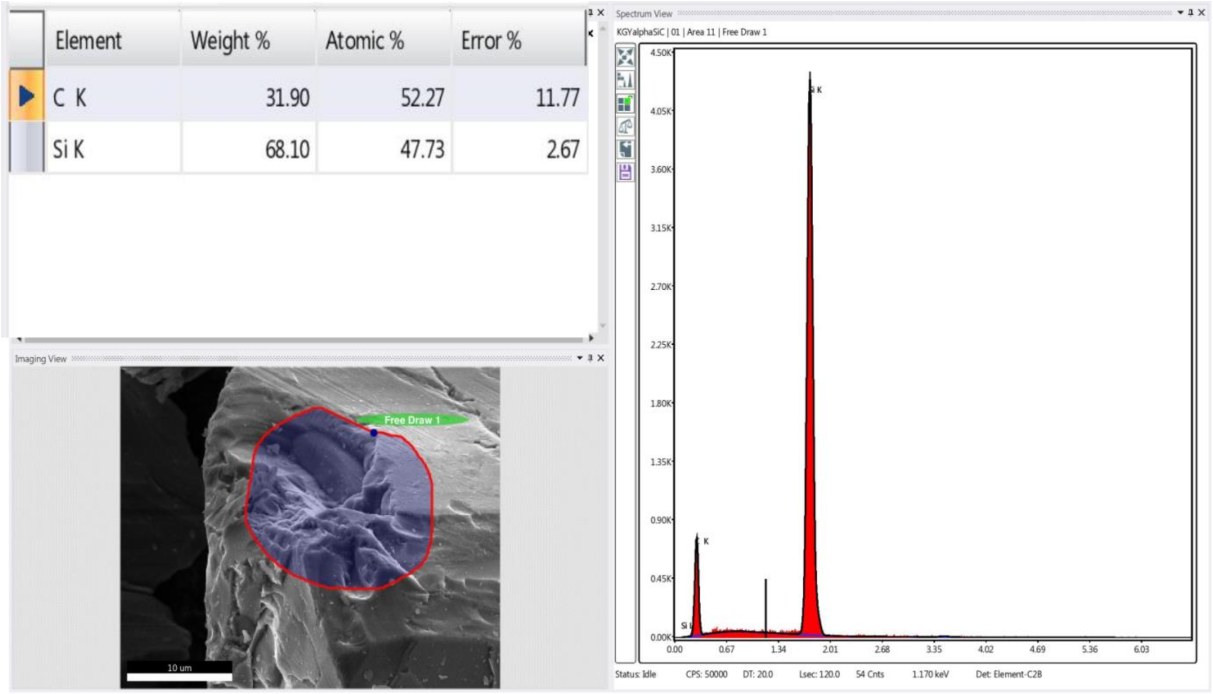

Figure S2. SEM-EDX of the bulk material after etching and sonicating.

Background correction for AFM and size distribution

First, we identified multiple large areas in the AFM pictures that contained no particles whatsoever, and drew dozens of profile lines to estimate the surface roughness based on the profile scattering and using statistical methods. Once the roughness has been estimated, we calculated the minimum height of a particle that could be distinguished from the surface. Then, we set the filters to exclude and eliminate particles that were undistinguishable from the surface. The remaning "dots" on the picture were considered to be real particles. These particles have been measured with two, orthogonal profile lines to determine the height of the particle which was later referred to as „size" of the particle. In both cases more than a hundred particle were measured for size distribution and the number was well above 300 for the retentate.

Estimation of the surface area and the pore depth

First, we provide an estimated size of our particles based on the SEM pictures using statistical methods. Measuring the particles resulted in the diameter of 140 ±80 μm. Taking 140 μm as average, for further simplification we are going to assume that the particles are sphere-shaped as spheres have the lowest possible surface to volume ratio. We also note that using cubes for modelling would lead to similar results and cubes are in better correlation with the SEM pictures while still having one of the lowest possible surface to volume ratio. Nevertheless the calculations demonstrated here were performed on the sphere model.

$d = 140\ \mu m = 0.014\ cm$

$A = 6.16 \times 10^{-4}\ cm^2$

$V = 1.44 \times 10^{-6}\ cm^3$

$m = 4.60 \times 10^{-6}\ g$

$A/V = 4.28 \times 10^{2}\ cm^2/cm^3$

$A/m = 1.34 \times 10^{2}\ cm^2/g$

Assuming that

- particles are sphere-shaped
- Performing etching followed by sonicating will remove a „porous shell" from this particle and will result in a sphere-shaped (or cube shaped) particle once again

we can give a lower estimation for the pore depth and for the surface as well, as in this model the first step (etching) increases the porosity and the second step (sonicating) decreases it.

For characterizing the etching rate we performed new measurements monitoring the weight of the samples using a high precision micro balance during the process. Using 2 hours and 4 hours of etching time the weightloss was 6% and 7%, respectively. These values are definitely results of the etching as the weight loss occuring during the sample transfer was also measured and it was below 5 mg (0.5%) in each cases. Sonicating these samples resulted in an additional 3% and 4% weightloss which is still a statistically significant weightloss.

The weightloss during the combined process, corrected by the weightloss coming from other sources can be estimated at 9%. Using the sphere model this means that the sphere has lost 3.1% of its diameter.

This model would suggest that the porosity depth is approximately 5μm.

After losing the porous shell to the sonicating and obtaining nanoparticles the surface to volume ratios can be calculated once again with the reduced diameter resulting

$A/V = 4.44 \times 10^2\ cm^2/cm^3$

$A/m = 1.39 \times 10^2\ cm^2/g$

which equals to 3.7% increasement in the surface after the sonication which is a rough lower estimate for the surface after etching and before the sonication.

We emphasize that this is a rough estimate but it is still on a par with the FIB-SEM pictures as they also portrait similar pore depth.

Detailed interpretation of the XPS spectra

It is known that due to the loss of photoelectrons during the photoemission process a positive charge will build up on the sample. As a result, the kinetic energy of the emitted photoelectrons will decrease resulting in a shift to higher values of binding energy of the observed peaks in the spectrum. First, we identified the adventitious carbon in the spectrum and shifted the spectrum by 3.9 eV so the peak position matched the literature data. Silicon carbide nanoparticles were dried on a Nb wafer and presumably the electric contact between the wafer and the semiconductor nanoparticles is inadequate. This causes a different charge for the SiC containing dried layer. This is confirmed e.g. by the presence of N 1s peaks with a difference of 1.4 eV. Assuming that these twin peaks emerge from the charged sample wafer and the insulating island of the nanoparticles simultaneously, an additional 1.4 eV shift was applied for the peaks associated with SiC. In case of the carbon spectrum it was not obvious which fitted components should be shifted by 3.9 eV and which ones should be shifted by 5.3 eV in total. We did a total permutation and managed to associate the fitted components to specified bonds as listed in **Table S1.**

Table S1. Fitted components and their associated bonds of the carbon spectrum

Binding energy (eV)	Chemical bond or group
284.5 eV	Adventitious carbon
289.8 eV	C=O group, C=S group
288.1 eV	C=O group
292.8 eV	C–F group

285.7 eV	C–C, C–H bond
282.8 eV	Si–C bond (shifted by 5.3 eV in total)
291.1 eV	CO_3^{2-} ion

Comparison of the infrared spectra of 3C and 6H SiC NPs

In order to observe the spectral change over time, droplets of the colloid sample were evaporated on a silicon wafer and the remaining material was measured by a Bruker Tensor 27 FTIR instrument in nitrogen atmosphere. Then the sample holder was heated to 180 °C.

Comparing the room temperature spectra of 6H-SiC NPs and 3C-SiC NPs (**Fig. S3.**) it can be concluded that the hydroxyl band centered at 3250 cm^{-1} is much broader in the cubic sample. This is related to the presence of the acidic carboxyl group which generally causes wider bands. The presence of the carbonyl band at 1720 cm^{-1} further supports this observation.

However, in case of the hexagonal sample there is no visible band in the 1700-1750 cm^{-1} region, and accordingly, the hydroxyl band has significantly smaller half width. This suggests that the surface is less acidic.

We also note that the hydroxyl band has little or no component at 3600-3750 cm^{-1}, which leads to the conclusion that silanol groups are not present in the sample. This is in good correlation with the XPS spectrum where no Si-O bonds were detected.

Additional changes were found upon heating the sample. Whereas the hydroxyl band of the 3C sample decreased over time while anhydride groups were formed [1], the hydroxyl band of the hexagonal sample increased over time, and other parts of the spectrum showed monotonous change as well (see **Figure S4. (a)-(e)).**

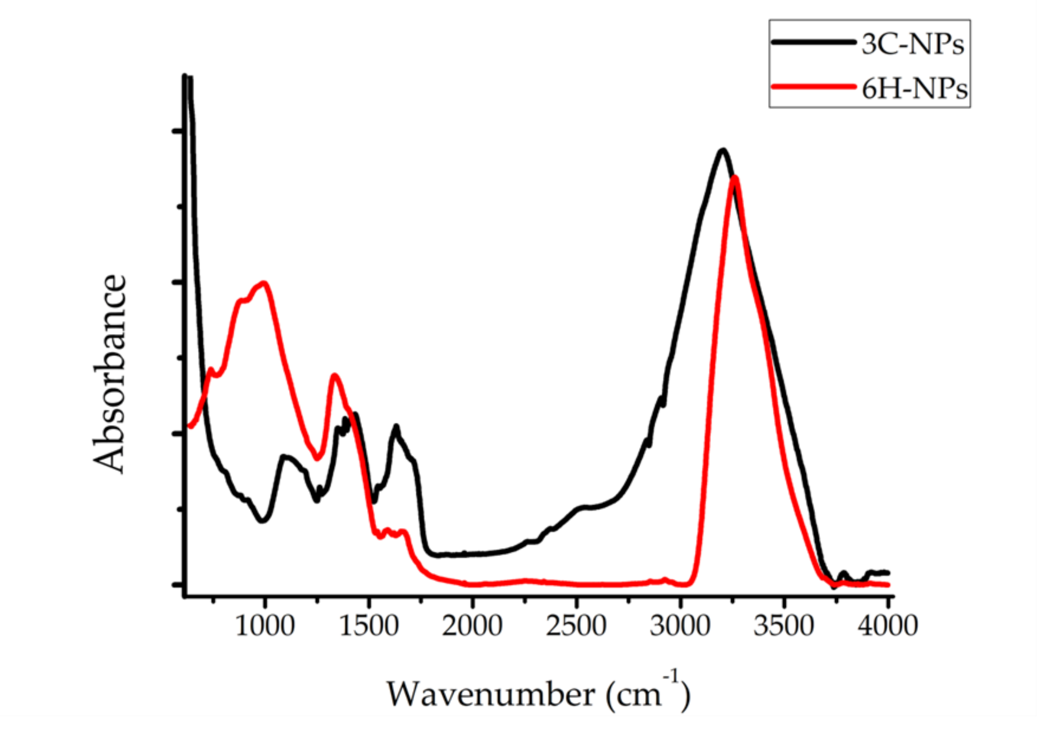

Figure S3. IR spectra of the 3C-SiC NPs and the 6H-SiC NPs. Spectra are baseline-corrected and shifted.

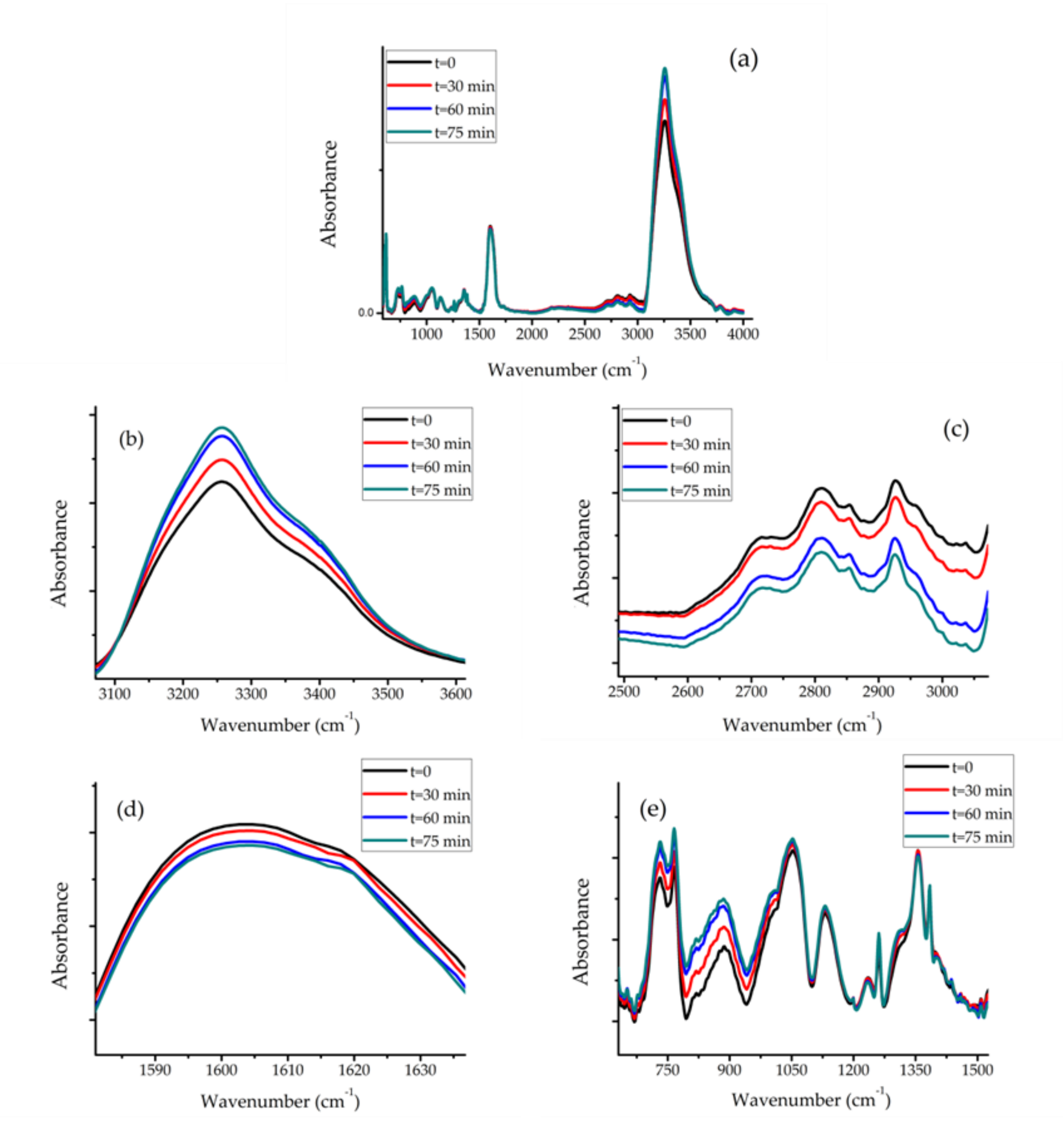

Figure S4. (a) The spectral change in the IR region of the 6H-SiC NPs over time at 180°C and **(b)-(e)** magnified parts of the **(a)** spectrum.

Szekrényes, Z., Somogyi, B., Beke, D., Károlyházy, Gy., Balogh, I., Kamarás, K., Gali, Á., Chemical Transformation of Carboxyl Groups on the Surface of Silicon Carbide Quantum Dots. *J. Phys. Chem. C* **2014**, 118, 19995–20001. https://doi.org/10.1021/jp5053024
